# Interdisciplinary management of mpox-related local complications: report on a series of cases

**DOI:** 10.3389/fmed.2023.1184924

**Published:** 2023-06-01

**Authors:** María Gamo Guerrero, Ana Simón Gozalbo, Mariángeles Martín Díaz, Kevin Díez Madueño, Emilio Del Río Pena, Pablo De la Cueva, Tamar Talaván, Eva Jiménez, Juan Torres, Jorge Valencia, Guillermo Cuevas, Carlos Bibiano, Pablo Ryan

**Affiliations:** ^1^Dermatology Service, Infanta Leonor Hospital, Madrid, Spain; ^2^Faculty of Medicine, Complutense University of Madrid, Madrid, Spain; ^3^Local Laboratory, Infanta Leonor Hospital, Madrid, Spain; ^4^Preventive Medicine Service, Infanta Leonor Hospital, Madrid, Spain; ^5^Internal Medicine Service, Infanta Leonor Hospital, Madrid, Spain; ^6^Infectious Diseases Unit, Internal Medicine Service, Infanta Leonor Hospital, Madrid, Spain; ^7^Emergency Service, Infanta Leonor Hospital, Madrid, Spain; ^8^Biomedical Network Research Centre on Infectious Diseases (CIBERINFECT), Madrid, Spain

**Keywords:** mpox, monkeypox, outbreak, complication, interdisciplinary, treatment, secondary infection

## Abstract

Monkeypox (mpox) is a viral zoonosis, and human-to-human transmission can result from close contact with the respiratory secretions and mucocutaneous lesions of an infected person. The prodromal phase is followed by an eruptive phase, with skin and/or mucosal lesions that progress through several stages at different sites. In this study, we describe the importance of interdisciplinary care management and follow-up of patients with complicated mpox. A cross-sectional study was conducted from May 2022 until August 2022 at a secondary hospital in Madrid (Spain). Out of 100 patients with mpox seen at this institution, we selected and analyzed 11 with local complications. All the patients were male at birth, and the mean age was 32 (30–42) years. The clinical manifestations included skin rash or mucosal lesions, fever, myalgia and lymphadenopathies. The most frequent local complications were pharyngitis associated with dysphagia, penile edema, infection of the mucocutaneous lesions, and ulceration of the genital lesions. A multidisciplinary team was created for the care of patients with complications secondary to mpox. The team comprised dermatologists and specialists in infectious diseases, preventive medicine, and emergency medicine. This approach improved the ability to diagnose and treat early with supportive, topical, and systemic treatment. In our center most of the cases were self-limiting, and none were life-threatening. An interdisciplinary response to a public health alert enhances the management of complex patients and should be implemented in successive outbreaks of mpox.

## 1. Introduction

The global outbreak of mpox was first recognized in the United Kingdom in May 2022 (1). Since then, many countries around the world have reported thousands of cases, especially in Europe and the USA, leading the WHO to declare an international public health emergency (2).

Mpox is caused by the monkeypox virus, a member of the *Orthopoxvirus* genus in the family Poxviridae (3, 4). The virus can be transmitted from human to human by close physical contact, such as kissing, skin-to-skin contact, and sexual relations. The current outbreak seems to have originated through sexual networks of men who have sex with men (MSM). This observation is supported by the fact that mpox is frequently accompanied by other sexually transmitted infections (STIs), with lesions in the genital or perianal area, and that it disproportionately affects people with multiple sexual partners (5).

The incubation period is usually 6 to 13 days, although this can range from 5 to 21 days (6). In the current outbreak, there is either no prodromal phase or the prodromal phase is short, with mild symptoms. These include fever, fatigue, headache, and localized lymphadenopathy and are accompanied or followed by an eruptive phase, with single or clustered mucocutaneous lesions that progress through several stages (macules, papules, pustules, and scabs) at different sites (mainly the oral cavity, fingers, and anogenital area [skin and mucosa]). They may also be accompanied by anal pain and odynophagia. The monkeypox virus causes mild, self-limiting disease (7–9), with a hospitalization rate of less than 10% and a case fatality rate of less than 1% (10). The disease appears to be more severe in patients with poorly controlled HIV infection characterized by a low CD4 cell count, especially when this is less than 200/mm^3^ (10–12). The most frequent complications in the present outbreak are cellulitis and infection of mucocutaneous lesions, rectal pain, penile edema, ocular involvement, and severe tonsillitis (5).

The antiviral agents authorized for treatment of mpox are tecovirimat and brincidofovir. However, insufficient efficacy data have been published for this outbreak, and the availability of tecovirimat is very limited (13–15). Therefore, the management of patients with mpox is oriented toward treatment and control of symptoms and prevention of possible complications. An interdisciplinary approach to this entity would enable complicated cases to be detected and treated earlier, thus effectively controlling the spread of the infection and ensuring that preventive public health measures such as isolation and case monitoring can be applied. In the present case series, we describe the characteristics, clinical features, and interdisciplinary management of patients with a complicated mpox.

## 2. Materials and methods

### 2.1. Study design

We conducted a longitudinal analysis of people with mpox confirmed by polymerase chain reaction (PCR) who were tested and managed at the Infanta Leonor Hospital in Madrid, Spain between May 2022 and August 2022. All patients signed an informed consent before inclusion in the study. The study protocol was approved by the Ethics Committee of Hospital Universitario Infanta Leonor and fulfilled the principles of the Declaration of Helsinki.

### 2.2. Interdisciplinary approach

**Figure 1:**
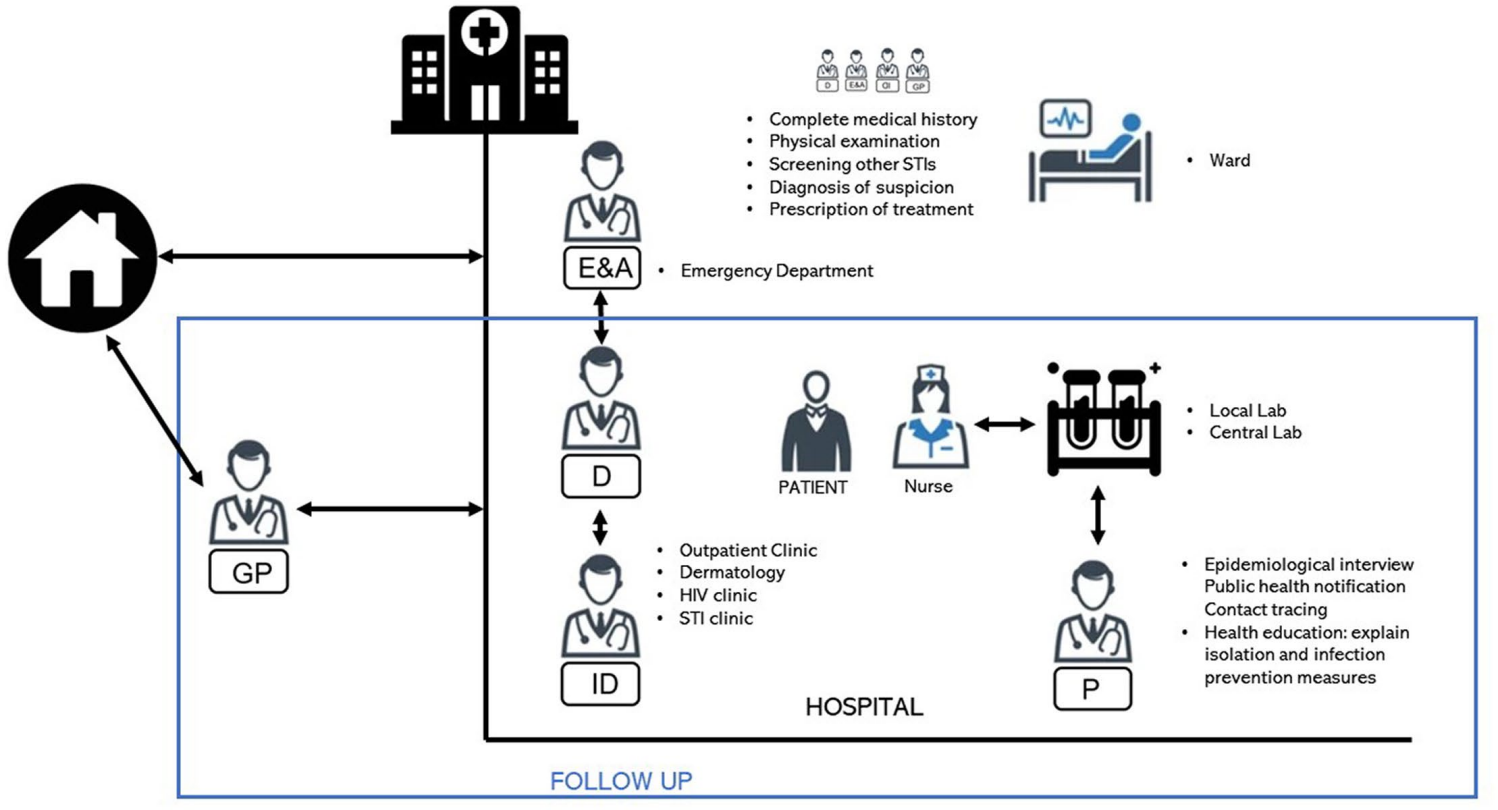
Patients were evaluated by an interdisciplinary team comprising dermatologists, nurses, and specialists in preventive medicine and infectious diseases. The team also includes personnel from the Public Health Department. GP, general practitioner; E&A, emergency medicine specialist; D, dermatologist; ID, infectious disease specialist; P, preventive medicine specialists.

Patients were evaluated by an interdisciplinary team comprising dermatologists, nurses, and specialists in preventive medicine, infectious diseases, and emergency medicine. The team also included personnel from the Public Health Department (Figure 1). Initially, patients were detected in the emergency department, primary care, or other specialties and referred to a dermatologist. The dermatologist worked together with an infectious disease specialist, who confirmed a suspected diagnosis, screened for other STIs, decided on the management of mucocutaneous lesions, and conducted a common follow-up, together with clinical sessions to discuss the management and progress of infected patients. Samples of the dermatological lesions and affected areas were then collected by a nurse and sent to the National Microbiology Centre for processing. Preventive medicine specialists informed patients about preventive and isolation measures, collected sociodemographic data through interviews, and reported the cases collected to the Public Health Department.

### 2.3. Data collection

The data collected included sociodemographic characteristics, clinical symptoms and signs reported at presentation, mucocutaneous manifestations, risk factors for HIV status, and the results of sexual health screening. In many cases, clinical photographs of lesions were also taken (after obtaining the patients’ informed consent). Study data were collected and managed using Microsoft Excel version 16.62 and the data capture tool Research Electronic Data Capture (REDCap) (16), which is hosted at “Asociación Ideas for Health.”

### 2.4. Statistical analysis

First, a descriptive analysis of data collected from all patients treated for mpox at the institution was conducted. A more in-depth analysis was then performed of those who experienced complications and/or a torpid clinical course from the infection. The sample was described using absolute and relative frequencies for categorical variables and median and interquartile range (IQR) for continuous variables. All analyses were performed using IBM SPSS Statistics for Windows, Version 21.0 (IBM Corp, Armonk, NY, USA).

## 3. Results

During the study period, 100 cases of mpox were confirmed based on PCR (samples from mucocutaneous lesions) at our institution. The median age was 33 years, 99 cases were male, and 60% had HIV infection. From all the patients with confirmed infection, we selected 11 (11%), who were characterized by an indolent course, slow recovery, local complications, and the need for interdisciplinary management and follow-up. The main characteristics of the individuals included in the study are shown in Table 1.

**Table 1:** Demographic and clinical characteristics of the patients with mpox in this case series.

	PATIENT 1	PATIENT 2	PATIENT 3	PATIENT 4	PATIENT 5	PATIENT 6	PATIENT 7	PATIENT 8	PATIENT 9	PATIENT 10	PATIENT 11
SEX	Male	Male	Male	Male	Male	Male	Male	Transgender female	Male	Male	Male
AGE (Years)	24	32	30	43	42	30	57	31	33	28	35
Sexual behavior	MSM	MSM	MSM	MSM	MSM	MSM	MSM	MSM	MSM	MSW	MSM
PREVIOUS STis	Syphilis	Syphilis	Neurosyphilis	Syphilis	Syphilis	Not reported	Not reported	Syphilis	Syphilis	Not reported	Syphilis
	Hepatitis B				Hepatitis B						GC urethritis
	Hepatitis A										CT proctitis
	CT urethritis										HPV condyloma
	ASCUS HPV										
HIV status	Positive on ART	Positive on ART	Positive on ART	Positive on ART	Positive on ART	Positive on ART	Positive on ART	Positive on ART	Positive on ART	Negative	Positive on ART
Recent sexual exposure	Yes	Yes	Yes	Yes	Yes	Yes	Yes	Yes	Yes	Yes	Yes
Recent trips abroad	No	No	No	No	No	No	No	No	No	No	No
Systemic symptoms	Fever	No	Fever	Fever	Fever	Fever	Myalgia	Fever	Myalgia	Fever	Odynophagia
	Myalgia		Myalgia		Myalgia	Myalgia	Odynophagia	Myalgia	Odynophagia		
	Asthenia		Asthenia		Asthenia	Asthenia		Asthenia			
					Anal pain			Odynophagia			
								Headache			
Lymphadenopathies	Painful inguinal nodes	Painful inguinal nodes	No	No	Painful inguinal nodes	Painful cervical nodes	Painful cervical nodes	Painful cervical nodes	Painful cervical and inguinal nodes	No	No
Location of skin lesions	Genital area	Genital area	Genital area	Genital area	Face	Genital area	Oral mucosa	Legs	Genital area	Genital area	Genital area
	Face		Face	Face	Back	Face	Arms	Arms	Pubic area	Pubic area	Oral mucosa
	Oral mucosa		Back		Legs	Legs	Legs	Thorax	Arms	Face	
	Thorax		Legs		Arms	Arms	Palm of hand	Abdomen	Legs	Neck	
	Back		Arms		Thorax	Pubic area		Face			
	Legs		Dorsum of hands		Anal area	Oral mucosa		Back			
	Arms				Abdomen			Oral mucosa (tonsillar hypertrophy)			
	Anal area				Sole of foot						
Hospital admission	Yes (4 days)	No	No	No	No	No	No	No	No	No	No
Treatment	Topical antibiotic (fusidic acid)	Topical antibiotic (fusidic acid)	Topical antibiotic (fusidic acid)	Topical antibiotic (fusidic acid)	Topical antibiotic (fusidic acid)	Topical antibiotic (fusidic acid)	Topical antibiotic (fusidic acid)	Topical antibiotic (fusidic acid)	Topical antibiotic (fusidic acid)	Topical antibiotic (fusidic acid)	Topical antibiotic (fusidic acid)
	Zinc dressing	Zinc dressing	Zinc dressing		Zinc dressing				Zinc dressing	Sodium borate dressing	
			Topical corticosteroid (betamethasone)	Topical corticosteroid (methylprednisolone)	Topical corticosteroid (betamethasone)	Topical corticosteroid (betamethasone)	Topical corticosteroid (betamethasone)			Topical corticosteroid (betamethasone)	Topical corticosteroid (betamethasone)
						Topical corticosteroid (triamcinolone acetonide 0.1% in orabase)	Topical corticosteroid (triamcinolone acetonide 0.1% in orabase)				
	Oral analgesic (paracetamol, ibuprofen)	Oral analgesic (paracetamol, ibuprofen)	Oral analgesic (paracetamol, ibuprofen)	Oral analgesic (paracetamol, ibuprofen)	Oral analgesic (paracetamol, ibuprofen)	Oral analgesic (Paracetamol, Ibuprofen)	Oral analgesic (paracetamol, ibuprofen)	Oral analgesic (paracetamol, ibuprofen)	Oral analgesic (paracetamol, ibuprofen)	Oral analgesic (paracetamol, ibuprofen)	Oral analgesic (paracetamol, ibuprofen)
	Oral antibiotic (ceftriaxone, azithromycin)	Oral antibiotic (ceftriaxone, azithromycin, penicillin)	Oral antibiotic (ceftriaxone, azithromycin, penicillin)	Oral antibiotic (amoxicillin-clavulanic acid 875/125 mg)	Oral antibiotic (amoxicillin-clavulanic acid 875/125 mg)			Oral antibiotic (amoxicillin-clavulanic acid 875/125 mg)	Oral antibiotic (amoxicillin-clavulanic acid 875/125 mg)	Oral antibiotic (amoxicillin-clavulanic acid 875/125 mg)	Oral antibiotic (amoxicillin-clavulanic acid 875/125 mg)
	IV antibiotic (amoxicillin-clavulanic acid 875/125 mg)			IV antibiotic (amoxicillin-clavulanic acid 2 g u.d.)							
Diagnostic test	Orthopoxvirus PCR	Orthopoxvirus PCR	Orthopoxvirus PCR	Orthopoxvirus PCR	Orthopoxvirus PCR	Orthopoxvirus PCR	Orthopoxvirus PCR	Orthopoxvirus PCR	Orthopoxvirus PCR	Orthopoxvirus PCR	Orthopoxvirus PCR
Time to hospital care (days)	2	3	9	4	6	7	5	1	4	2	2
Complication	Superinfection	Ulceration	Ulceration	Cellulitis	Cellulitis	Ulceration	Pharyngitis	Tonsillitis	Ulceration	Ulceration	Ulceration
	Ulceration									Penile edema	Penile edema

MSM, men who have sex with men; CT, *Chlamydia trachomatis*; ASCUS, atypical squamous cells of undetermined significance; ART, antiretroviral therapy; GC, gonococcal.

**Figure 2:**
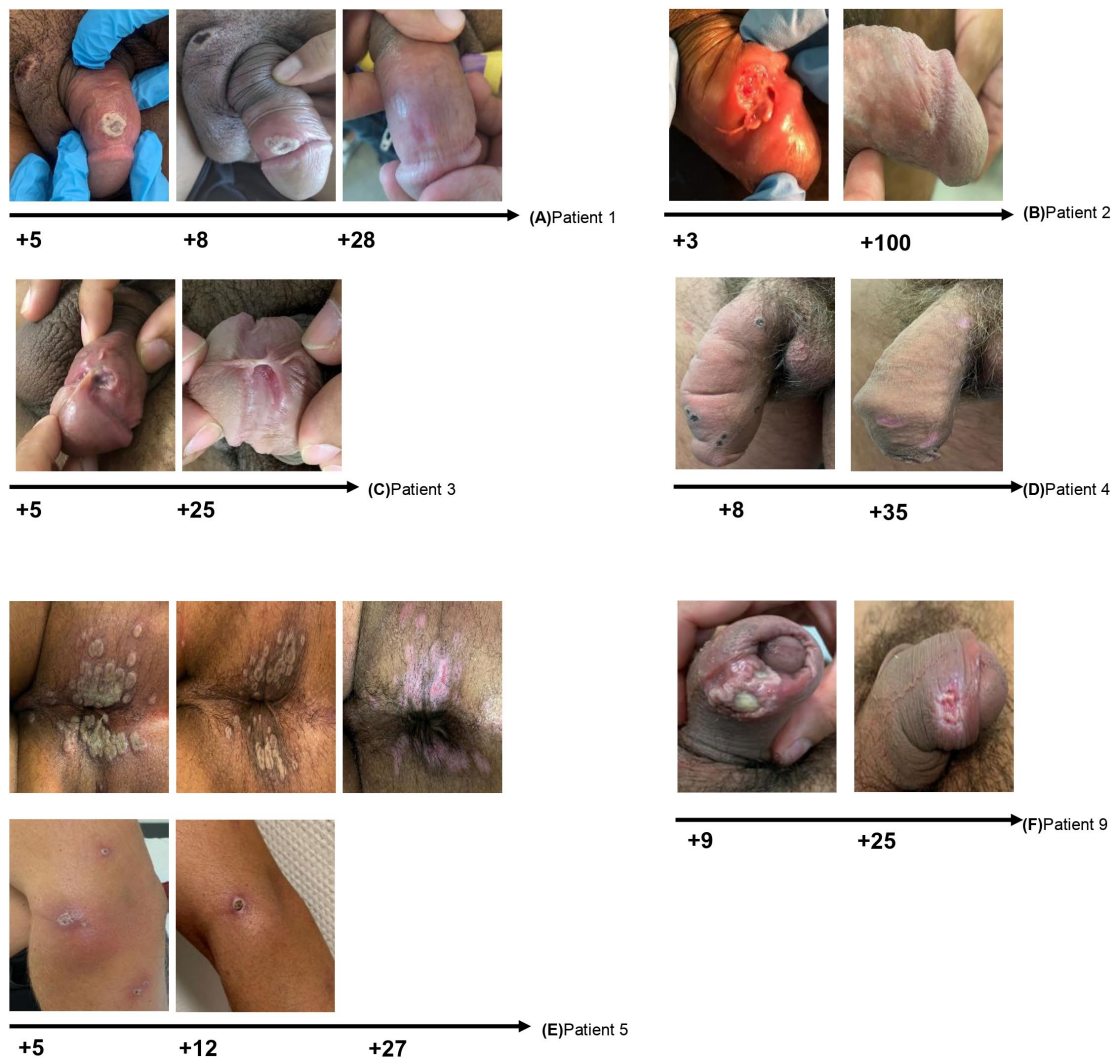
Clinical course of local complications in patients with mpox. The main complications were secondary infection, ulceration of genital lesions, penile edema, and cellulitis. Progress was very favorable after the first month, although most lesions left scars. The deeper lesions persisted with a visible defect after 4 weeks.

**Figure 3:**
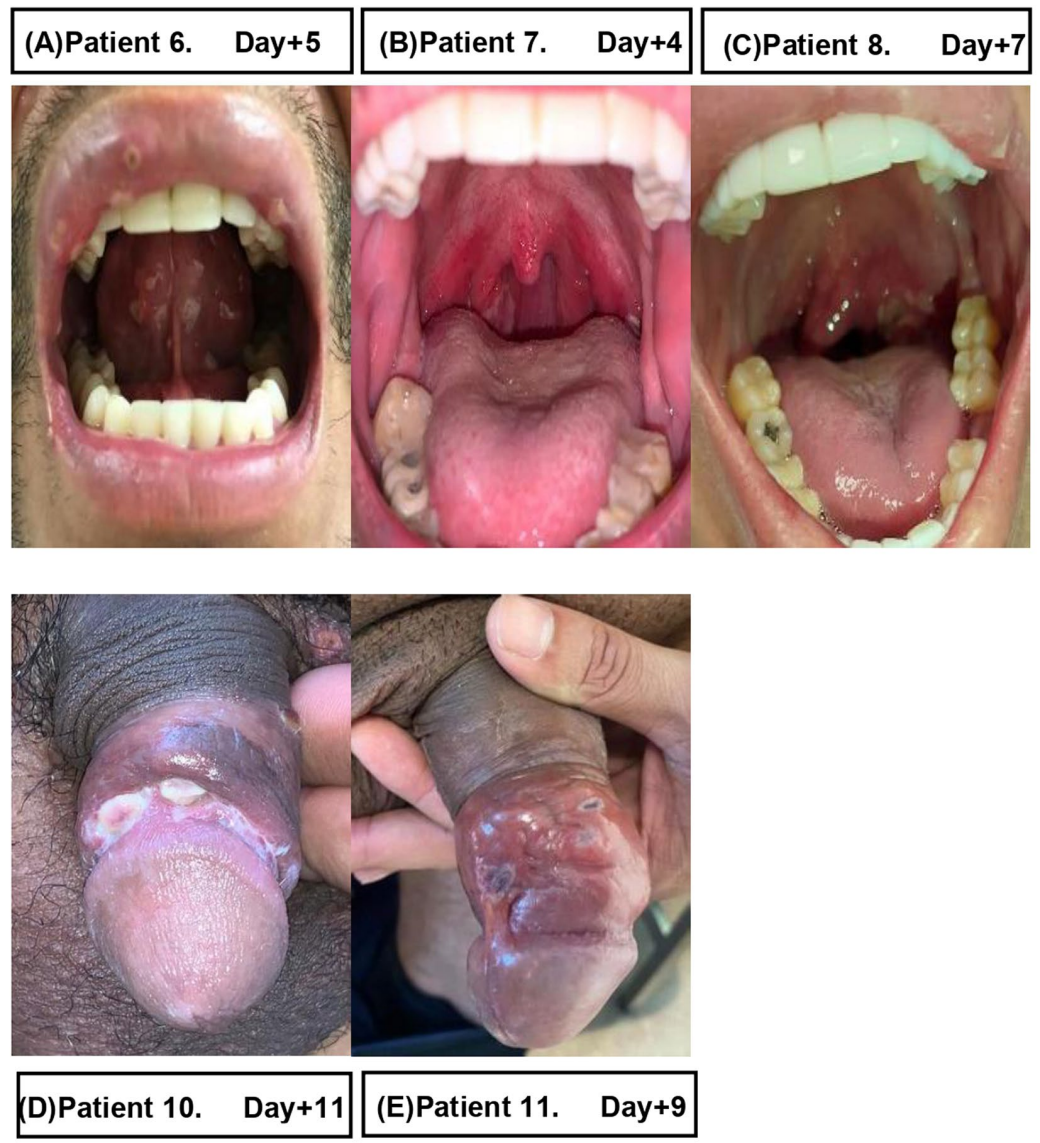
Local complications of patients with mpox and days from symptom onset. Patient 6 **(A)** presented very painful lesions on the oral mucosa. Patient 7 **(B)** presented pharyngitis and lesions at the level of the palate. Patient 8 **(C)** presented tonsillitis with severe tonsillar inflammation and deviation of the uvula accompanied by intense odynophagia. Patients 10 **(D)** and 11 **(E)** developed ulceration of the genital lesions accompanied by significant edema, with difficulty retracting the foreskin in the case of patient 11 **(E)**.

The median age was 32 (30–42) years. All the patients were male at birth; one was a transgender woman. Ten patients were males who had sex with males and HIV-infected (91% of our sample). The only person who did not engage in same sex practices was not HIV-infected. All the HIV-infected patients were receiving antiretroviral therapy (ART) and had an undetectable HIV viral load. In relation to previous risk behaviors and STI, none of the patients had a regular partner, all had had unprotected sex in the previous weeks, and 7/10 had previously had an STI other than HIV (mainly syphilis and urethritis). Patients were seen at the emergency department and the outpatient clinic. Patients sought health care a median of 4 (IQR 2–6) days after onset of symptoms. Ninety percent of the patients presented with general symptoms before the cutaneous rash, including fever (64%), myalgia (55%), fatigue (45%), headache (9%), and pharyngitis (9%), accompanied in 64% of cases by painful lymphadenopathies located mainly in the inguinal area (36%) and in the cervical area (27%). The progress of the mucocutaneous lesions was asynchronous, developing in multiple stages, with some of them forming scabs and crusts, while others remained in the early papular, vesicular, and pustular phases. The most commonly affected anatomical sites were the genital area (73%), legs or arms (64%), face (55%), oral mucosa (45%), thorax or back (27%), anal or pubic area (18%), and the palm and dorsum of the hand, sole of the foot, and abdomen (9%). Depending on the location, these findings were accompanied by symptoms such as odynophagia (36%) and anal pain (18%; Figures 2, 3).

Treatment was aimed at controlling symptoms and management of complications. All the patients received oral analgesia for pain management (mainly acetaminophen and/or NSAIDs). The skin and/or mucosal lesions were treated with topical antibiotic therapy (fusidic acid) and topical corticosteroids (betamethasone or methylprednisolone). Triamcinolone acetonide 0.1% was prescribed to patients with lesions affecting the oral mucosa. In addition, zinc or sodium borate dressings were recommended in half of the patients.

The most common complication was ulceration of genital lesions, sometimes with significant edema and difficulty retracting the foreskin (Figures 2, 3). Scarring was observed after 1 month (Figure 2) and fibrosis after 3 months [Figure 2, patient 2 (B)]. One patient developed cellulitis of the arm and ulceration of perianal lesions, although these had almost resolved after 1 week of antibiotic treatment, resulting in a visible scar 1 month later [Figure 2, patient 5 (E)]. Oral or intravenous antibiotic therapy was required for those with slowly progressing lesions or concomitant STIs (Table 1). Other common complications included painful ulcers in the oral mucosa with pharyngitis and tonsillitis, odynophagia [Figure 3, patient 6,7,8 (A, B, C)]. All patients were pain-free and asymptomatic after 4 weeks.

Of the 100 patients diagnosed with mpox at our institution, 6 (6%) were hospitalized. However, only 1 of them was admitted for complications (infection of the ulcerative lesions and cellulitis), which required intravenous antibiotics and analgesics. The reasons for the remaining admissions included concomitant infections other than mpox (eg, COVID) and the impossibility of home isolation. No ICU admissions or deaths due to mpox were reported during the study period.

Figure 1 illustrates the interdisciplinary team brought together for the joint management of mpox cases during this global outbreak. Some of the tasks included collecting biological samples to confirm mpox or to rule out STI, reporting cases to the Public Health Department, conducting epidemiological surveys, performing blood tests, implementing isolation recommendations, creating local protocols, delivering systemic and local treatments, and monitoring of patient progress at the clinic or by telephone. The follow-up was carried out in the outpatient clinics, with close monitoring of the patients’ clinical evolution and dermatological lesions. All of those involved worked together to address the multiple physical and psychological needs of individual cases and to implement an integrated approach to treatment.

## 4. Discussion

We report a series of 11 cases from among 100 patients with a confirmed diagnosis of mpox at our institution (11%). The course of the disease was indolent, and management required a multidisciplinary approach. Our findings confirm the characteristics of the current outbreak of mpox in non-endemic countries—mainly affecting males who have sex with males infected via sexual transmission—and the complications described are similar to those reported in the literature (5, 11, 12, 17).

The most common complications in our series were ulceration of genital lesions, which results in scarring and fibrosis 3 months later. Cellulitis and infection of mucocutaneous lesions, rectal pain, penile edema, pharyngitis, and tonsillitis were the most frequent complications. Complications due to bacterial infection improved with prompt identification and early antibiotic therapy. After 1 month, none of the patients had any related symptoms. The complications recorded were similar to those reported elsewhere (10, 18, 19).

We did not record any serious complications or deaths related to the infection.

Consistent with previously reported results in mpox cases, most patients had a history of STIs, frequent risky sexual practices, sporadic partners, and unprotected sex. In this sense, mpox patients should be screened for STIs, as is usual for any other sexually transmitted pathogen (20). It has been suggested that underlying immune deficiencies may lead to worse outcomes in mpox (11, 12, 21, 22). Out of the 100 patients with confirmed mpox attended at our hospital, 60% were HIV-positive. These patients were admitted more frequently (0% vs. 8%) and had more frequent risk behaviors (unprotected sex, chemsex, multiple sexual partners) than patients without HIV infection. Of those with complicated mpox, 91% were HIV-positive and receiving ART. Although most of the patients in our case series were HIV-positive, our results do not allow us to state that the course of mpox disease is more severe in HIV-positive patients. In fact, in the literature published to date, the clinical course of individuals with well-controlled HIV infection is very similar to that of individuals without HIV. Nevertheless, patients with uncontrolled HIV and CD4 cell counts below 200 cells per mm^3^ have been reported to develop severe, disseminated form of mpox with 15% mortality (11, 12, 23). No one with well-controlled HIV and no one who received a mpox vaccine died, which suggests that starting and staying on antiretroviral therapy can prevent severe outcomes.

All patients with confirmed mpox were managed by an interdisciplinary team that was created as a response to the health alert in May 2022. According to Morse and Schluederberg (24), the use of interdisciplinary teams is essential for the early detection, containment, and management of emerging infectious diseases and their public health consequences. Furthermore, the article notes that effective coordination and communication between different disciplines are critical for ensuring an effective response to infectious diseases. Our interdisciplinary approach involved team members from different disciplines working together (nurses, infectious diseases physicians, dermatologists, specialists in preventive medicine and public health, laboratory staff, and emergency department staff). It was a comprehensive patient care strategy that aimed to improve communication with patients and provide a linear trajectory of care.

In our experience, the team set goals, made decisions, and shared resources and responsibilities in the management infected patients. This circuit allowed the discussion and problem-solving of cases from different angles, with early detection of cases and same-day referrals to dermatology or infectious diseases. One of the key components of this circuit was the processing and recording of samples and laboratory results for STIs and mpox, using a centralized approach that involved nursing, laboratory, and the preventive service. Furthermore, having a reference team within the hospital to which patients could be referred, and accelerating diagnostic and care circuits, along with the discussion of complex cases, were critical components of this interdisciplinary model.

Most patients in our series were treated on an outpatient basis, with only 1 requiring hospital admission. As in other reports, treatment was empirical. Care was optimized to relieve symptoms, control complications, and prevent long-term sequelae and was based mainly on oral and topical antibiotic therapy and topical corticosteroid therapy (25, 26). Patient progress was favorable in the short and medium terms. Secondary bacterial infections were treated as indicated: 82% received oral and/or iv antibiotic therapy and 100% received topical antibiotic therapy (Table 1). Amoxicillin-clavulanic acid was used as the first-line option because it is an orally available antibiotic with a broad spectrum of action. In patients with pharyngitis or tonsillitis, it was essential to optimize analgesic treatment to improve odynophagia. Furthermore, topical corticosteroids were applied in ulcerated lesions with a significant inflammatory component, mainly medium-potency corticosteroids, such as betamethasone or methylprednisolone. In 2022, based on human and animal studies, the European Medicines Agency (EMA) authorized the antiviral tecovirimat, originally developed for smallpox, for treatment of mpox (27) Although we did not have access to tecovirimat, our results were favorable, with minimal short- and long-term sequelae. Approval was based on human and animal studies, although efficacy, safety, and clinical practice data for antivirals in mpox are limited (28) Further studies are needed to assess the efficacy of these agents on disease course and severity and prevention of infection.

The SARS-COV2 pandemic highlighted the need for a multidisciplinary approach in healthcare settings. This new challenge confirms the importance of an interdisciplinary approach to health emergencies. The next step will be the development of diagnostic and therapeutic protocols and algorithms to improve management practices for this and other challenging conditions.

## 5. Conclusion

Mild-to-moderate complications are common in patients with mpox. In this case series, we observed that if adequate treatment is managed by an interdisciplinary team, improvement is rapid, sequelae are minimal, and management can be on an outpatient basis.

## Data availability statement

The original contributions presented in the study are included in the article/supplementary material. Further inquiries can be made to the corresponding author.

## Ethics statement

The studies involving human participants were reviewed and approved by Ethics Committee of Hospital Universitario Infanta Leonor. The patients/participants provided their written informed consent to participate in this study. Written informed consent was obtained from the individual(s) for the publication of any potentially identifiable images or data included in this article.

## Author contributions

MG and PR: conceptualization, investigation, writing – original draft preparation, and writing – review and editing. PR: formal analysis and supervision. All authors contributed to the article and approved the submitted version.

## Funding

This study was supported by grants from Instituto de Salud Carlos III (Centro de Investigación Biomédica en Red en Enfermedades Infecciosas; CB21/13/00044).

## Conflict of interest

The authors declare that the research was conducted in the absence of any commercial or financial relationships that could be construed as a potential conflict of interest.

## Publisher’s note

All claims expressed in this article are solely those of the authors and do not necessarily represent those of their affiliated organizations, or those of the publisher, the editors and the reviewers. Any product that may be evaluated in this article, or claim that may be made by its manufacturer, is not guaranteed or endorsed by the publisher.
